# Changes in plant species composition of coastal dune habitats over a 20-year period

**DOI:** 10.1093/aobpla/plv018

**Published:** 2015-04-15

**Authors:** Silvia Del Vecchio, Irene Prisco, Alicia T. R. Acosta, Angela Stanisci

**Affiliations:** 1 Centre for Estuarine and Marine Studies, DAIS, Università Ca’ Foscari Venezia, Castello 2737b, 30122 Venezia, Italy; 2 Dipartimento di Scienze, Università degli Studi Roma Tre, V.le Marconi 446, 00146 Roma, Italy; 3 Dipartimento Bioscienze e Territorio, Università degli Studi del Molise, Via Duca degli Abruzzi, 86039 Termoli (CB), Italy

**Keywords:** Coastal dune zonation, diachronic analysis, phytosociological relevés, re-visitation study, vegetation changes

## Abstract

Coastal sandy ecosystems are increasingly being threatened by human pressure, causing loss of biodiversity, habitat degradation and landscape modifications. However, there are still very few detailed studies focussing on compositional changes in coastal dune plant communities over time. In this work, we investigated how coastal dune European Union (EU) habitats (from pioneer annual beach communities to Mediterranean scrubs on the landward fixed dunes) have changed during the last 20 years. Using phytosociological relevés conducted in 1989–90 and in 2010–12, we investigated changes in floristic composition over time. We then compared plant cover and the proportion of ruderal, alien and habitat diagnostic species (‘focal species’) in the two periods. Finally, we used Ellenberg indicator values to define the ‘preferences’ of the plant species for temperature and moisture. We found that only fore dune habitats showed significant differences in species cover between the two time periods, with higher plant cover in the more recent relevés and a significant increase in thermophilic species. Although previous studies have demonstrated consistent habitat loss in this area, we observed that all coastal dune plant communities remain well represented, after a 20-year period. However, fore dunes have been experiencing significant compositional changes. Although we cannot confirm whether the observed changes are strictly related to climatic changes, to human pressure or to both, we hypothesize that a moderate increment in average yearly temperature may have promoted the increase in plant cover and the spread of thermophilic species. Thus, even though human activities are major driving forces of change in coastal dune vegetation, at the community scale climatic factors may also play important roles. Our study draws on re-visitation studies which appear to constitute a powerful tool for the assessment of the conservation status of EU habitats.

## Abstract (precis)

Coastal sandy ecosystems are increasingly being threatened by human pressure, causing loss of biodiversity and habitat degradation. Using phytosociological relevés we conducted a re-visitation study in order to analyse changes in floristic composition during the last twenty years along the central Adriatic coast. We observed a significant increase in cover of fore dune and thermophilic species. Even though human activities are major driving forces of change in coastal dune vegetation, the species' cover increase may also be due to a moderate increment in average yearly temperature over the last two decades.

## Introduction

Coastal sandy ecosystems are currently among the most threatened ecosystems (EEA 2008). Several studies have emphasized the various stages of coastal dune deterioration throughout Europe, as well as highlighting increasingly threatening human pressure (e.g. Heslenfeld *et al*. 2004; Schlacher *et al*. 2008; Drius *et al*. 2013). In fact, human activities in coastal areas have intensified over the course of the 20th century (Defeo *et al*. 2009; Feola *et al*. 2011; Romano and Zullo 2014). Ever-increasing tourism, the expansion of urban areas and the spread of agriculture and afforestation activities have strongly modified coastal landscapes (Alados *et al*. 2004; Hesp and Martínez 2007). Climate may also be an important driver of vegetation composition and plant community structure (Bruelheide 2003; Kreyling *et al*. 2008; Wang *et al*. 2013). Many studies indicate that temperature and rainfall regimes have experienced variation due to global changes coupled with rapid population growth and urbanization (Brunetti *et al*. 2006; Diffenbaugh *et al*. 2008; Carrete *et al*. 2009). The major direct ecological effect of global change on coastal ecosystems is the lengthening of the vegetative season, which may facilitate the spread of thermophilic species, both natives and aliens (Sobrino Vesperinas *et al*. 2001; UNEP 2010; Provoost *et al*. 2011), although an increase in phytomass has also been observed along North European coasts (Jones *et al*. 2013).

A previous study demonstrated that coastal habitats show the highest level of risk and require further research into the changes in vegetation at both the landscape and community scale (La Posta *et al*. 2008). However, these habitats have often been neglected in such analyses, since coastal dune systems are often overlooked in medium- and large-scale studies and are ignored in local and regional planning (Carboni *et al*. 2009).

Recently, efforts have been made to analyse trends in coastal land cover types over time. Malavasi *et al*. (2013) evaluated changes in coastal dune spatial patterns over the last 50 years using land cover maps derived from a multi-temporal sequence of remotely sensed data. These authors emphasized that the composition and structure of coastal landscapes have been drastically modified by human activities. In particular, from the post war period until the present day, the loss of natural coastal dune habitats has occurred together with the expansion of artificial areas, afforestation and the gain of new land for agricultural activities. In contrast, compositional changes in coastal dune plant communities over time have not yet been explored, such analysis remaining an important but difficult research task since floristic information for previous decades is often scarce.

Europe has a long tradition of vegetation surveys based on the classical phytosociological approach (Braun-Blanquet 1964; Westhoff and van der Maarel 1973; Dierschke 1994; Dengler *et al*. 2008). This has proved a very useful methodological framework, not only for local and regional overviews of vegetation types (Schaminée *et al*. 2009), but also for thorough analyses of vegetation changes over time (Jandt *et al*. 2011; Jantsch *et al*. 2013; Chytrý *et al*. 2014). In Italy, a huge number of phytosociological relevés have recently been collected in national vegetation databases (Landucci *et al*. 2012; Prisco *et al*. 2012). In particular, for most vegetation types, there is a lack of detailed floristic information obtained in previous decades to compare with more recent relevés at a local scale. Along the Italian Adriatic coast, many relevés were sampled in dune habitats during the late 1980s; thus, now 20 years later, a re-visitation study was conducted using the same field protocol and at the same sites.

On that basis and in order to take advantage of having comprehensive floristic information for one area surveyed twice through the phytosociological approach (Braun-Blanquet 1964), in the present study we investigated how the vegetation of the coastal dunes has changed over 20 years. We compared plant species composition and cover using phytosociological relevés carried out in 1989–90 with relevés carried out in 2010–12. Furthermore, as indicators of the changes in vegetation, we analysed variations in the proportions of ruderal and alien species and the habitat's diagnostic species (‘focal species’). Finally, we used Ellenberg indicator values to define the ‘preferences’ of the plant species for a certain temperature and moisture regime, analysing whether the communities responded with a variation in these preferences. We assumed that Ellenberg indicator values, when derived from the mean values of several species in conjunction, provide reliable and easily calculated proxies for environmental factors when actual empirical measurements are missing (Lawesson *et al*. 2003).

## Methods

### Study area

**Figure 1.**
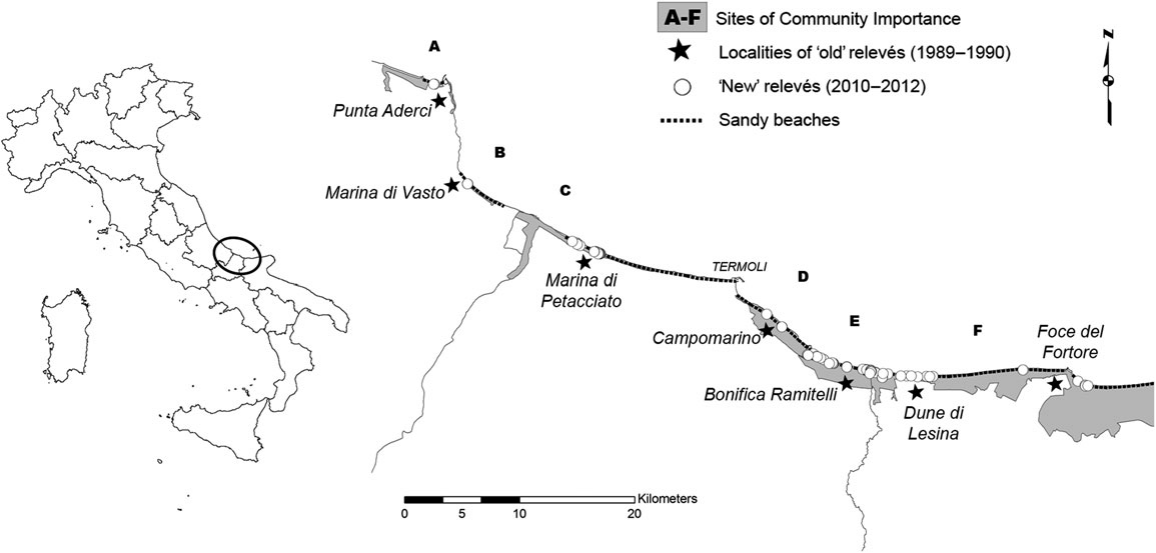
Distribution of the relevés along the coast of the Abruzzo, Molise and Apulia regions. Sites of Community Importance are shown in grey. Black stars identify the sampling localities of relevés conducted in 1989–90, whereas white circles show the relevés conducted in 2010–11. The black dotted line indicates segments of sandy beaches along the shoreline.

**Figure 2.**
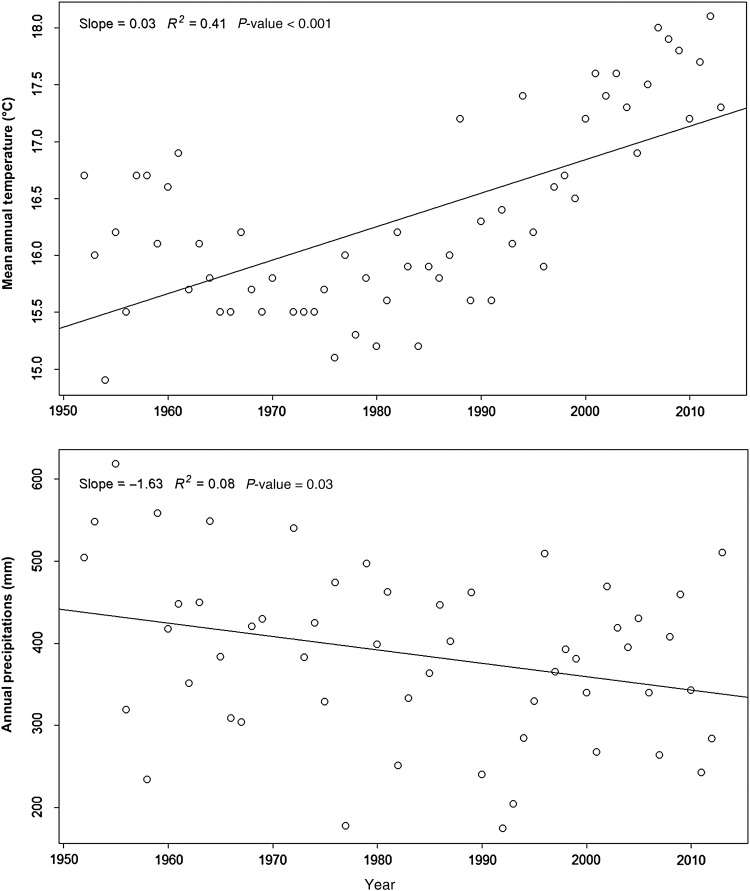
Analysis of the mean annual temperature and precipitation time series from 1950 to the present day.

The study area stretches for ∼70 km along the Adriatic Sea, comprising the Abruzzo, Molise and Apulia regions (Fig. 1); it is mainly composed of sandy beaches. The area includes six sites of community importance (SCIs): (A) Punta Aderci—Punta della Penna (IT 7140108), (B) Marina di Vasto (IT7140109), (C) Foce Trigno—Marina di Petacciato (IT7228221), (D) Foce Biferno—Litorale di Campomarino (IT7222216), (E) Foce Saccione—Bonifica Ramitelli (IT7222217) and (F) Dune e Lago di Lesina—Foce del Fortore (IT9110015) (Fig. 1). In this area, recent dunes (Holocene) occupy a narrow strip along the seashore. These dunes are not very high (<10 m height) and they are relatively simple in structure (usually only one dune ridge) (Acosta *et al*. 2009). As well as the dune profile, abiotic conditions vary greatly along the sea-inland gradient, leading to habitat zonation. Under natural conditions, the vegetation zonation follows this ecological gradient, ranging from pioneer annual communities on the beach to Mediterranean scrubs on the landward fixed dunes. The mean annual temperature in Termoli (climatic station in the middle of our study area) is 16.3 °C and the mean yearly precipitation amounts to 385.8 mm (data available at http://www.scia.isprambiente.it/home_new.asp, referring to the 1950–2013 period). On the basis of the SCIA climatic database (Desiato *et al*. 2006, 2007, 2011), which includes climatic data from specific stations, we analysed the variation in temperature and precipitation in the study area (Termoli station) over the last 60 years. In particular, we evaluated changes in yearly time series of mean temperature and annual precipitation from 1950 up to present using a general linear model (R statistical software, R Core Team 2014). This climatic analysis highlighted a significant increase in the mean annual temperature (slope: 0.03, *P*-value: <0.001) coupled with a significant decrease in the annual precipitation (slope: −1.63, *P*-value: 0.03) (Fig. 2).

### Data collection

We collected 87 phytosociological relevés conducted in 1989–90 from a literature review (Taffetani and Biondi 1989; Stanisci and Conti 1990; Pirone *et al*. 2001). We selected only those relevés occurring in relatively stable dune systems (Aucelli *et al*. 2004; Miccadei *et al*. 2011) and accompanied by an accurate description of the localities. During 2010–12, we re-visited the same areas and performed 71 new phytosociological relevés (Table 1). Since no permanent plots were marked in the first sampling period, during the 2010–12 field work activity we re-visited the same area following the description of the location reported in the reference studies. In particular, relevés were conducted following the same sampling protocols (considering plant community type, plot size, previous species lists and dominant species cover estimations) (Chytrý *et al*. 2014) and in the same season in order to remove effects of phenological differences (Vymazalová *et al*. 2012). In addition, in order to limit the pseudo-turnover caused by observer bias (Klimeš *et al*. 2001; Vittoz and Guisan 2007), one of the researchers who conducted some of the 1989–90 sampling was also involved in the 2010–12 field work activity. During 2010–12, we were able to geo-reference each relevé with relatively high geographic accuracy using a GPS unit. Each relevé was then assigned to a European Union (EU) habitat type following the guidelines of the Italian Interpretation Manual of the 92/43/EEC Habitats Directive (Biondi *et al*. 2009) and the Interpretation Manual of European Union Habitats (European Commission 2013). We pooled these habitats into four groups: drift line (habitat 1210), fore dune (habitat 2110 and 2120), dune grasslands (habitat 2230) and fixed dune (habitat 2250 and 2260) (Table 1). Sampling size varied according to the habitat type (2–100 m^2^), but was the same within each habitat. We used Conti *et al*. (2005) as a taxonomic reference list. Cases of synonymy and taxonomic problems (see Jansen and Dengler 2010) were resolved using the Conti *et al*.'s updated list of synonyms.

**Table 1.** List of habitats and relevés information. For each habitat category analysed is reported the Habitat Directive code, the name, a brief description, the number of relevés considered in each time interval, the localization in the Sites of Community Importance and the reference source for the old relevés.

Habitat			Number of relevés			Sites of Community Importance	References
EU code	Name	Description	Total	1989–90	2010–12		
1210	Drift line	Formations of annuals occupying accumulations of drift material	17	8	9	IT7140109, IT7228221, IT7222216	Taffetani and Biondi (1989), Stanisci and Conti (1990)
2110, 2120	Fore dune	First formations of sand accumulation and mobile dunes forming the seaward cordon	78	47	31	IT7140108, IT7140109, IT7228221, IT7222216	Taffetani and Biondi (1989), Stanisci and Conti (1990), Pirone *et al*. (2001)
2230	Dune grasslands	Associations of many small annuals with abundant ephemeral spring bloom	22	11	11	IT7140109, IT7228221	Stanisci and Conti (1990), Pirone *et al*. (2001)
2250, 2260	Fixed dune	Juniper formations and sclerophyllous scrubs of Mediterranean coastal dune slacks	42	21	21	IT7228221, IT7222217, IT9110015	Taffetani and Biondi (1989)

The plant communities were sampled using the classic phytosociological approach. We recorded the list of vascular plant species identified within each plot and the percentage of cover of each species, using the Braun-Blanquet scale of abundance/dominance (Braun-Blanquet 1964; Westhoff and van der Maarel 1973). For each relevé, we totalled the percentage cover of each species; thus, this parameter can exceed 100. Moreover, we calculated the percentage cover of focal species, alien species and ruderal species (grouping alien and ruderal species in a single guild). We chose these species guilds because previous studies on coastal dunes demonstrated that focal species are reliable indicators of adequate conservation state and of proper community functioning, whereas aliens and ruderals are associated with disturbance (Carboni *et al*. 2011; French *et al*. 2011; Del Vecchio *et al*. 2013). Moreover, we assigned to each species the Ellenberg indicator value for temperature and moisture, and calculated the means in each relevé. Although restrictions should be applied, various studies have shown that average indicator values can be considered an effective way to relate vegetation change to environmental changes (Pignatti 2005; Jantsch *et al*. 2013). On the basis of the method introduced by Ellenberg *et al*. (1992) for the German flora, Pignatti (2005) proposed the same indicators adapted for the Italian vascular flora. In particular, the scale of the indicators for temperature and light was extended from 9 to 12, so as to include the warmer and brighter conditions of the Mediterranean relative to the conditions in continental Europe ones. Therefore, for the specific purposes of this study, we defined ‘thermophilic’ species as those with Ellenberg temperature values higher than 8.

### Data analyses

We analysed a matrix of 131 species × 158 relevés via detrended correspondence analyses (DCA) using the R statistical software (R Core Team 2014—Vegan package; Oksanen *et al*. 2013). Then, we performed an analysis of similarities through a one-way analysis of similiarity (ANOSIM) test (9999 permutations) to search for significant differences between groups of relevés, depending on the year in which they were carried out (Past software; Hammer *et al*. 2001).

For each habitat group, we compared total species cover and the frequency of focal, alien and ruderal species in the relevés carried out in 1989–90 and 2010–12. In addition, for each relevé we calculated the mean Ellenberg indicator values of temperature and moisture weighted on species cover. We checked for gross violations of normality using the Shapiro–Wilk *W* test (Shapiro and Wilk 1965) and visual estimation of the data distribution. Non-normally distributed data were square root transformed. We performed a permutational multivariate analysis of variance (PERMANOVA, 9999 randomizations), including the effect of the year (factor with two levels) and the habitat type (factor with four levels) as grouping variables. We also included the interaction between year and habitat type, allowing us to test whether the effect of year varied by habitat. Finally, the post hoc Tukey HSD test was performed on ranked data to investigate which means contributed to the observed effect (Past software; Hammer *et al*. 2001).

## Results

**Figure 3.**
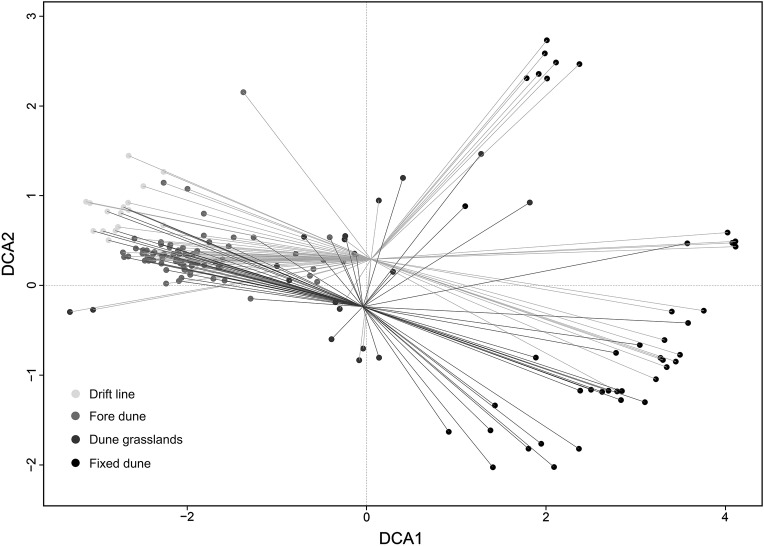
Detrended correspondence analyses scatter diagram of plots (grouped in the four habitat types), using species as explanatory variables. Only the first two axes are represented. Light grey lines represent the relevés sampled in 1989–90; dark grey lines represent the relevés sampled in 2010–12.

Eigenvalues for the DCA axes were 0.887 for axis 1 (DCA1) and 0.578 for axis 2 (DCA2). As expected, the first axis primarily reflected the strong coastal dune vegetation zonation along the sea-inland environmental gradient, ranging from the drift line to the fixed dune habitats (Fig. 3). Meanwhile, the second axis revealed differences in the floristic composition of the relevés, depending on the date they were sampled. In particular, the ordination scatter diagram separated the relevés into two groups, one corresponding to the relevés conducted in 1989–90 (the upper group) and the other corresponding to the relevés conducted in 2010–12 (the lower group) (Fig. 3).

The analysis of similarity supported these results. The ANOSIM test revealed a significant difference between the relevés carried out in the past and in the present (ANOSIM *R*-value = 0.025; *P* = 0.039).

**Figure 4.**
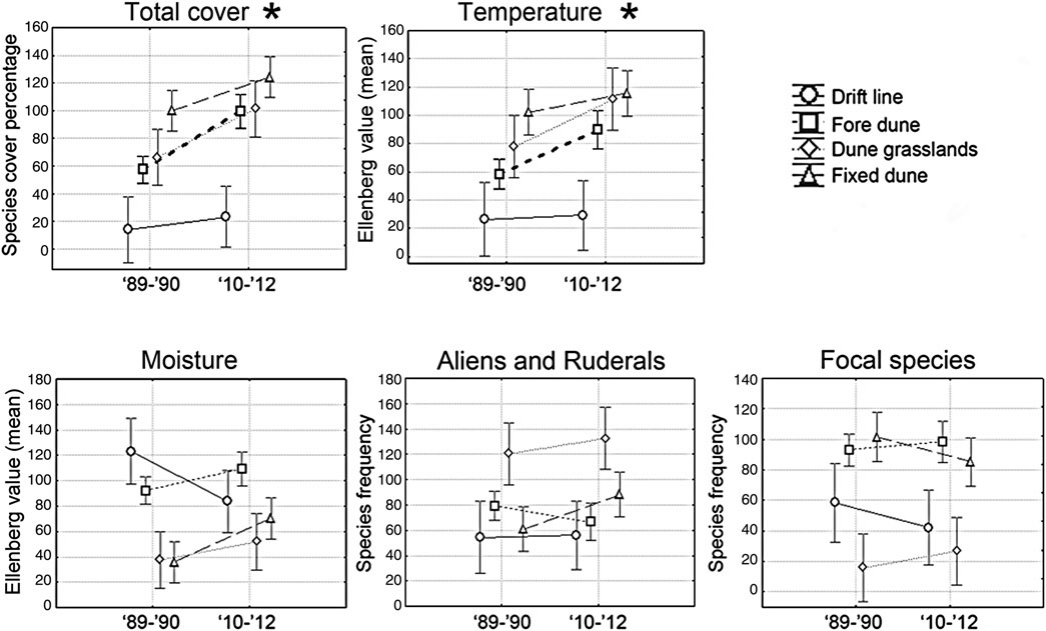
Comparison of species cover, mean Ellenberg indicator values for temperature and moisture, and species guild frequency between the relevés sampled in 1989–90 and 2010–12 by habitat type. Vertical bars denote 0.95 confidence intervals. The black stars and the thick dashed lines indicate significant differences found using the post hoc Tukey HSD tests (*P* < 0.05).

The PERMANOVA test revealed effects of the habitat group, the year and their interaction on the dependent variables (Table 2). Specifically, the Tukey HSD test showed differences in the percentage of species cover between the two temporal groups with higher percentages of plant cover in the more recent relevés, albeit these differences were significant only for the fore dune habitat (Fig. 4).

**Table 2.** Permutational multivariate analysis of variance (PERMANOVA) result. Effect of the year and the habitat group on species cover, mean Ellenberg indicator values for temperature and moisture, focal species and alien and ruderal species. Asterisks indicate significant results.

Source	Sum of squares	df	Mean square	*F*	*P*
Year	0.14676	1	0.14676	7.4365	0.0001***
Habitat group	1.469	3	0.48967	24.811	0.0001***
Interaction	−0.71871	3	−0.23957	−12.139	0.0489**
Residual	2.9603	150	0.019736		
Total	3.8574	157

**Figure 5.**
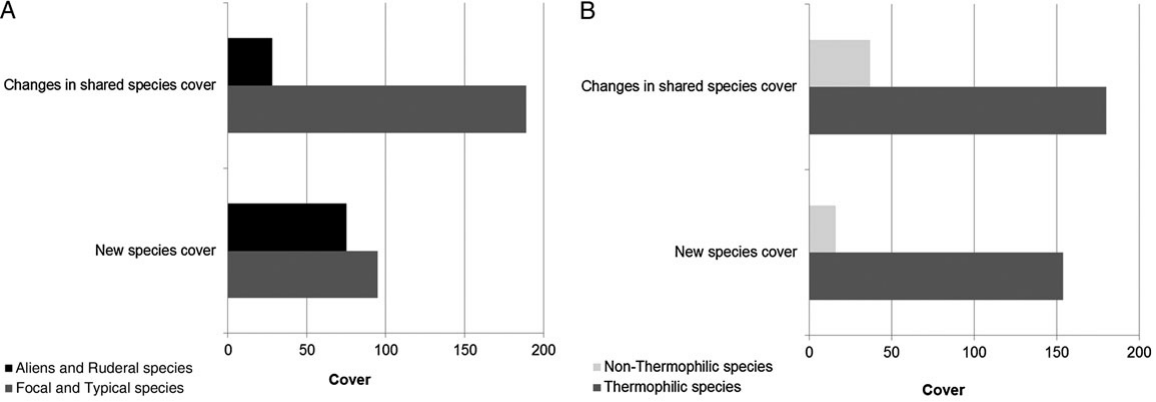
Change in plant cover and species composition in fore dune vegetation over 20 years. (A) Comparison between total cover of focal/typical dune species and alien/ruderal species. (B) Comparison between total cover of thermophilic species (Ellenberg *T* value >8) and non-thermophilic species (Ellenberg *T* value <8). In both cases, the change in shared species cover was calculated as differences in plant cover for the two time intervals (1989–90 and 2010–12). New species cover refers to those only found in the 2010–12 relevés.

In particular, some focal and typical fore dune species [e.g. *Lotus creticus* L., *Calystegia soldanella* (L.) Roem. & Schult., *Elymus farctus* (Viv.) Runemark ex Melderis and *Ammophila arenaria* (L.) Link) together with some ruderal species (e.g. *Reichardia picroides* (L.) Roth and *Sixalix atropurpurea* (L.) Greuter & Burdet subsp. *grandiflora* (Scop.) Soldano & F. Conti] increased their cover. Moreover, new focal species were found in the recent relevés (e.g. *Sporobolus virginicus* Kunth, *Anthemis maritima* L. and *Pancratium maritimum* L.) along with other typical dune species [e.g. *Sonchus bulbosus* (L.) N. Kilian & Greuter, *Medicago littoralis* Loisel. and *Polygonum maritimum* L.] (Fig. 5A).

Regarding Ellenberg values, we observed an increase in the mean indicator value for temperature in almost all habitats between 1989–90 and 2010–12 (Fig. 4). However, we should note that significant differences were observed only for the fore dune habitat. In particular, the spreading thermophilic species were mainly typical fore dune species [e.g. *Calystegia soldanella* (L.) Roem. & Schult., *Lotus creticus* L.], but there were also some ruderals [*Reichardia picroides* (L.) Roth and *Sixalix atropurpurea* (L.) Greuter & Burdet subsp. *grandiflora* (Scop.) Soldano & F. Conti]. Moreover, among the new arrivals, many species were also thermophilic, including the typical fore dune species *Anthemis maritima* L., *Sporobolus virginicus* Kunth, *Pancratium maritimum* L., the ruderals *Calendula arvensis* L., *Hypochaeris achyrophorus* L., *Polypogon maritimus* Willd. and other psammophilous species [e.g. *Medicago littoralis* Loisel., *Sonchus bulbosus* (L.) N. Kilian & Greuter, *Polygonum maritimum* L., *Hedypnois rhagadioloides* (L.) F.W. Schmidt and *Ambrosia maritima* L.] (Fig. 5B). However, changes in focal, alien and ruderal species and the Ellenberg values for moisture were not significant.

## Discussion

Comparison of the phytosociological relevés conducted 20 years apart, revealed that all sand dune plant communities detected in the relevés from 1989 to 90 are still well represented in the relevés from 2010 to 12. Although previous studies indicate consistent habitat loss in this area (Malavasi *et al*. 2013; Romano and Zullo 2014) and only a few sites along the Italian Adriatic coast have preserved their high plant community richness (Frattaroli *et al*. 2007; Sburlino *et al*. 2008; Prisco *et al*. 2013), the presence of all coastal habitats previously identified is an encouraging result, suggesting the discrete conservation status of dune ecosystems in the study area.

Detailed analyses of the changes in species cover and composition showed significant increments in the total plant cover and in the frequency of thermophilic species. Similar trends have been documented in other European coastal ecosystems over the last few decades and have been mainly related to the effects of global climate change (Sobrino Vesperinas *et al*. 2001; Provoost *et al*. 2011; Jones *et al*. 2013). Thus, even though previous studies affirmed that human activities are major driving forces of change in coastal dune vegetation at the community scale (Malavasi *et al*. 2014), climatic factors may also play important roles. In fact, climatic changes may act as important drivers in vegetation composition and plant community structure due to direct physiological species responses (caused by the variation in nutrient quantities, temperature range and water availability) or to indirect species responses (caused by alterations in biotic interactions, such as competition) (Bruelheide 2003; Isbell *et al*. 2013). Our results showed that perennial thermophilic focal species contributed the most to the increase in recorded plant cover. Some of these species were already present in the older relevés, and others, such as *Anthemis maritima* L., *Sporobolus virginicus* Kunth and *Pancratium maritimum* L., were more common only along the Tyrrhenian sandy coast (Stanisci *et al*. 2004).

Although similar ecological processes were detectable in all the investigated dune habitats, only fore dunes showed significant changes. We hypothesize that the moderate increase in average yearly temperature observed may have promoted the increase in plant cover and the spread of thermophilic plant species that previously grew mainly along the warmer Tyrrhenian and Ionian coasts. Floristic changes in fore dunes, dominated by rhizomatous grasses such as *Ammophila arenaria* (L.) Link and *Elymus farctus* (Viv.) Runemark ex Melderis, are particularly important because they are likely the most important habitats on sandy coasts due to their role in preventing coastal erosion, in mitigating flooding and maintaining and enhancing the natural, cultural and amenity values of beaches (Drius *et al*. 2013; Stoll *et al*. 2015).

Re-visitation studies are challenging. Even though we are confident that the new sampling was conducted in the same plant communities as the historical sampling, the results might have suffered some bias due to a possible mismatch. Moreover, it is worth highlighting that, based on our results, we cannot affirm whether the observed changes in coastal dune species cover and composition were strictly related to climatic changes, to human pressure or to both. However, this work is a preliminary step, demonstrating that coastal dune plant communities have experienced significant compositional changes during the past 20 years. These changes may have important implications for biodiversity conservation, as well as for long-term predictions of the effects of global climate change (Heijmans *et al*. 2008). Further studies focussing on the assessment of recent vegetation changes should be conducted to develop a better understanding of coastal dune ecosystem dynamics. Re-visitation studies comparing historical phytosociological relevés and newly resampled vegetation plots may prove a powerful tool for assessing vegetation changes, although detailed monitoring studies are also required for accurate evaluation of temporal trends.

## Sources of Funding

This work was partially supported by Life+ project EnvEurope—Environmental quality and pressures assessment across Europe: the LTER network as an integrated and shared system for ecosystem monitoring (http://www.enveurope.eu/) under grant number LIFE08 ENV/IT/000399 for the re-survey of 2010–12.

## Contributions by the Authors

A.S. and A.T.R.A. conceived and designed the experiments. S.D.V. and I.P. analysed the data. S.D.V., I.P., A.T.R.A. and A.S. wrote the manuscript.

## Conflict of Interest Statement

None declared.
